# Responsiveness of the ten-metre walk test, Step Test and Motor Assessment Scale in inpatient care after stroke

**DOI:** 10.1186/1471-2377-14-129

**Published:** 2014-06-16

**Authors:** Katharine Scrivener, Karl Schurr, Catherine Sherrington

**Affiliations:** 1 Musculoskeletal Division, The George Institute of Global Health, The University of Sydney, PO Box M201 Missenden Road, Sydney 2050 NSW, Australia; 2 Department of Health Professions, Macquarie University, Sydney, Australia; 3 Physiotherapy Department, Bankstown-Lidcombe Hospital, Sydney, Australia

**Keywords:** Stroke, Responsiveness, Sensitivity, Motor performance, Measurement

## Abstract

**Background:**

Responsiveness of a measurement tool is its ability to detect change over time. The aim of this study was to determine the responsiveness and floor/ceiling effects of the ten-metre walk test (10mWT), Step Test and Motor Assessment Scale (MAS) lower limb items.

**Methods:**

An inception cohort study was conducted, including 190 stroke survivors admitted to a comprehensive stroke unit. The 10mWT, Step Test and MAS were administered within 48 hours of admission and repeated in the 48 hours before discharge. Responsiveness was analysed with Effect Size (ES), Standardised Response Mean (SRM) and a median-based Effect Size (mES). Floor/ceiling effects were calculated as the percentage of participants scoring the lowest/highest possible scores.

**Results:**

Responsiveness of each outcome measure varied according to the statistic used. Values for the 10mWT were ES 1.44, SRM 0.93, mES 0.45; the step test ES 1.99, SRM 0.88, mES 0.36; MAS sit-to-stand (item 4) score ES 1.27, SRM 1.00, mES 0.50; and for MAS item 5 (walking) ES 1.43, SRM 1.10, mES 0.50. The MAS item 3 (sitting balance) was moderately responsive in all analyses (ES 0.72, SRM 0.71, mES 0.50). The MAS mobility score (summed items 3-5) consistently showed large responsiveness (ES 1.42, SRM 1.16, mES 0.92). The Step Test had the highest proportion of participants who didn’t change (46%) and item 4 of the MAS showed the largest ceiling effect on discharge (44%).

**Conclusions:**

Most measures were able to detect change in motor performance during inpatient stroke rehabilitation but the MAS mobility score was the only measure that demonstrated large responsiveness and no marked floor or ceiling effects.

## Background

A common goal of rehabilitation after stroke is to improve motor performance. A variety of tools to measure stroke survivors’ motor performance are available. Clinicians and researchers need to choose measurement tools that are appropriate to their setting. Factors to consider when assessing the usefulness of a measurement tool for a particular setting include, reliability, validity, responsiveness and floor or ceiling effects [1,2]. The current literature provides little information to guide clinicians in the choice of measurement tools in an inpatient setting. Measures including the ten-metre walk test (10mWT), Step Test and Motor Assessment Scale have been chosen for routine use on the study stroke unit so we sought to assess their responsiveness in the context of usual post-stroke intervention.

Responsiveness refers to a measurement tool’s ability to detect change over time in a specific population [1]. Responsiveness of tools that measure aspects of motor performance after stroke has not been as thoroughly investigated as have reliability and validity [3]. To date, most of the studies of responsiveness of these tools have a number of limitations. Many studies have involved small sample sizes e.g. 16 to 35 participants, and used samples of convenience rather than consecutive samples of stroke survivors [4]. Other studies have significant exclusion criteria e.g. excluding stroke survivors based on their physical abilities on admission [4], making their samples unrepresentative of the true post stroke population. The measurement tools previously found to be highly responsive in an inpatient setting are the six-minute walk test (Standardised Response Mean [SRM] 1.52), twelve-minute walk test (SRM 1.90) and the Rivermead Mobility Index (Effect Size [ES] = 1.28) [4].

Floor and ceiling effects may limit a measurement tool’s ability to detect change in a particular population. In other words, if a significant proportion of the population have scores at the bottom (floor) or top (ceiling) of the range of possible scores [5], then the tool will not necessarily measure change in performance.

In this study we investigated the responsiveness of the ten-metre walk test (10mWT), Step Test and lower limb items of the Motor Assessment Scale and examined the distribution of collected data for floor and ceiling effects. The ten-metre walk test was first described by Wade and colleagues in 1987 [6], since then it has become a common clinical measure of gait speed used in rehabilitation. The subject is asked to walk a 14 m track as quickly but safely as possible and the middle 10 m is timed. This measure has been shown to be a reliable and valid measurement tool in stroke survivors (inter-tester reliability, intraclass correlation coefficient [ICC] = 0.89-90; test-retest reliability, ICC 0.87-0.99) [3]. In a recent systematic review of the responsiveness of measurement tools during inpatient care after stroke, the ten-metre walk test demonstrated variable responsiveness with effect sizes of 0.55 and 1.17 [4]. Furthermore, in inpatient stroke rehabilitation, a floor effect at admission has been identified with 31% of participants scoring 0 m/s (i.e., unable to walk) [7].

The Step Test involves the subject attempting to place his/her foot continuously on and off a 7.5 cm block as quickly as possible, without losing balance, in a 15 second time period. The test was introduced by Hill in 1996 and shown to be a reliable and valid measure of balance after stroke (test-retest reliability, ICC > 0.88) [8]. To our knowledge the responsiveness of the Step Test has not previously been investigated in the stroke population.

The Motor Assessment Scale was developed and reported by Carr and Shepherd in 1985 [9]. It is a comprehensive measure of motor performance that involves rating the subject’s performance of common functional tasks such as sitting and walking. There are eight items included in the scale and two items relate to bed mobility, three to lower limb functional tasks (balanced sitting, moving from sitting to standing and walking) and three to upper limb function. Each item is scored on a scale ranging from zero to six. In a recent systematic review of the responsiveness of measurement tools during inpatient care after stroke, the lower limb items of the MAS had variable responsiveness with effect sizes ranging from 0.61 to 1.03 [4]. The item that appears most responsive to change is item 5 (walking) [4]. A floor effect on admission to stroke rehabilitation has been identified for item 5 (walking, 39% of participants scoring lowest score) [7]. A ceiling effect on admission to and discharge from stroke rehabilitation for item 3 (balanced sitting, 57% and 60-92% of participants respectively scoring highest score) [7,10] and a ceiling effect on discharge for item 4 (sitting to standing, 54-62%) [7,10]. In this study we examined MAS items 3 (balanced sitting), 4 (sitting to standing) and 5 (walking) individually and scores for these items were also summed (MAS mobility score). We included 190 consecutive stroke survivors who participated in physiotherapy. The stroke survivors were assessed at the beginning and end of their admission to a comprehensive stroke unit, consisting of both acute and rehabilitation phases of care.

The research questions were:

1. How responsive are the 10mWT, Step Test and lower limb items of the MAS to change in performance during inpatient care after stroke?

2. What proportion of participants did not change their scores for each measurement tool?

3. Are there any floor or ceiling effects in the measures at admission or discharge?

## Methods

An inception cohort study was completed on the Bankstown-Lidcombe Hospital stroke unit [11]. All people after stroke all people in this hospital are admitted to the stroke unit. The stroke unit is a comprehensive unit with co-located acute and rehabilitation beds, we were able to observe stroke survivors throughout the acute and rehabilitation phases of their hospital stay. There is no delineation between the acute and rehabilitation phases of care with all stroke survivors offered intensive rehabilitation from early after their admission to the unit. The Human Research Ethics Committee of the Sydney South West Area Health Service approved this study (project number QA2008/049).

As this is a busy government hospital the stroke unit can also have medical patients with any diagnosis admitted for a temporary period. It is usual practice at this site for people after minor strokes or transient ischemic attacks to be admitted to the unit for assessment and medical management. Therefore, all people admitted to the unit with a diagnosis of stroke and requiring physiotherapy intervention were considered for inclusion in the study. People were excluded from the study if their primary diagnosis was not stroke, or they had minor symptoms that did not require inpatient physiotherapy or they were admitted for palliation after the stroke.

The treating therapist measured participants on the 10mWT, Step Test and scored items 3–5 of the MAS within 48 hours of admission to the unit. Participants were retested on these measures during the 48 hours prior to discharge. All assessors were provided with an assessment protocol and standard equipment. The first author provided training in assessment methods and ongoing feedback regarding the accuracy of each assessor’s adherence to the protocol. The measures chosen were routinely administered on the stroke unit.

On weekdays participants undertook an intensive physiotherapy program that focussed on task-specific training of motor skills such as sitting, sit-to-stand, standing and walking [12,13]. In addition, strength training was completed in both weight-bearing and non weight-bearing positions. On average participants completed 288 (SD 240, range 1–1136) lower limb exercise repetitions on each day of therapy [14].

### Outcome measures

#### Ten-metre walk test

To conduct the 10mWT in this study the subject was asked to walk as fast as possible, but safely, along a 14 m track. The middle 10 m were timed. If the subject was unable to walk 10 m the speed for the maximal distance was recorded. If he/she was unable to walk at all, the velocity was recorded as 0 m/s.

#### Step Test

To conduct the test in this study a 7.5 cm high block was placed 5 cm in front of the subject’s feet. The subject’s base of support was allowed to be no wider than the block (30 cm). The subject was asked to place the whole foot on and off the block as fast as possible, without losing balance, continuously for 15secs. The best of three attempts on each leg was recorded. If he/she required steadying the number of completed steps prior to the steadying was recorded. If he/she was unable to step without assistance a score of 0 was recorded. The poorer side was used for data analysis.

#### Motor Assessment Scale

In this study the Motor Assessment Scale items 3–5 were completed using standard instructions and equipment [13].

### Data analysis

Data analysis was completed in SPSS. The MAS scores for items 3 to 5 were summed at admission and discharge to form an MAS mobility score. The summing of MAS upper limb item scores has been previously researched [15]. To our knowledge the summing of other MAS items has not been investigated to date.

Responsiveness was assessed as described by Husted and colleagues [16]. We calculated Effect Size I (Cohen’s Effect Size) (ES) by taking the mean baseline score minus the mean follow-up score divided by standard deviation of baseline scores. We calculated the Effect Size II (Standardise Response Mean [SRM], Responsiveness-Treatment coefficient, efficiency index) by taking the mean baseline score minus mean follow-up score divided by standard deviation of the change from baseline to follow up. As the outcome measures investigated included an ordinal scale (the MAS) and/or had skewed distributions, a median-based Effect Size (mES) was also calculated by dividing the median change score by the 30th to 70th inter-percentile range (Q3/7) of the change score [17]. The interpretation of Effect Sizes was described by Cohen in 1977 as small effect size is 0.2, moderate effect size 0.5 and large effect size 0.8 [18]. We operationalized this definition to mean that a small Effect Size, SRM and mES was between 0.2 and 0.49, a moderate Effect Size was between 0.5 and 0.79 and a large effect sixe was 0.8 or larger. The alpha level of significance used was 0.05.

We quantified the proportion of participants whose score on each measurement tool did not change between admission and discharge.

In order to investigate each measure’s possible floor or ceiling effects we used descriptive statistics (frequency of each score) for admission and discharge. Each score was graphed as a histogram and the distribution of scores was inspected. We considered a poor result to be more than 20% of participants scoring the minimum (floor) or maximum (ceiling) score [19].

## Results

From January 2008 to April 2010, 1014 people were admitted to the stroke unit. Two hundred people met the inclusion criteria for the study and were assessed within 48 hours of admission. People were excluded from the study if their primary diagnosis was not stroke (425 people) or because they had a TIA, minor stroke, or were admitted for palliation after stroke and did not require inpatient physiotherapy (389 people). Of the included participants, 7 died during their admission. Three participants did not have complete admission or discharge data available for analysis and were excluded from the study. Consequently data for 190 participants were included in the analysis.

The characteristics of the 190 included participants on admission to the stroke unit are described in Table 1. The stay on the unit varied with a range of 10 to 81 days of therapy, and an average of 22.5 therapy days (SD 13.3) or approximately four weeks. On discharge, 69% of participants returned home, 24% were discharged to an aged care facility and 7% were transferred to another hospital or ward.

**Table 1:** Characteristics of study participants on admission, n = 190

**Characteristic**	**Study population**
Age (mean, SD)	76.0, 12.7
Sex (female n,%)	93, 49%
Type of stroke	
L CVA (n,%)	72, 38%
R CVA (n,%)	80, 42%
Haemorrhagic (n,%)	23,12%
Cerebellar/brainstem (n,%)	13, 7%
Other (n,%)	2, 1%
Modified Rankin Score (mean, SD)	4.2, 0.77
Charlston Co-morbidity Index (mean, SD)	1.6, 2.0

### Responsiveness

The responsiveness of each measurement tool is shown in Table 2. Responsiveness for the 10mWT varied from ES 1.44, SRM 0.93 to mES 0.45. The Step Test was highly variable dependant on the method of analysis with an ES of 1.99, SRM 0.88 and mES of 0.36. Responsiveness for MAS item 3 was ESI 0.72, SRM 0.71, mES 0.50, MAS items 4 was ES 1.27, SRM 1.00, mES 0.50 and MAS item 5 was ES 1.43, SRM 1.10, mES 0.50. The summed MAS item 3–5 showed the most consistent and large responsiveness with ES 1.42, SRM 1.16, mES 0.92.

**Table 2:** Summary of the responsiveness and floor and ceiling effects of motor performance measures in stroke rehabilitation, n = 190

**Measure**	**Admission, mean (SD)**	**Discharge, mean (SD)**	**ES**	**SRM**	**mES**	**Number (%) participants that did not change**	**Floor effect, number (%)**		**Ceiling effect, number (%)**	
							**Admission**	**Discharge**	**Admission**	**Discharge**
	**Median (IQR)**	**Median (IQR)**								
Step Test	0.65 (2.2)	4.94 (5.4)	1.99	0.88	0.36	87 (45.8)	166 (87.4)	87 (45.8)	N/A	N/A
	0.00 (0.0)	3.00 (9.0)								
Walking velocity (10mWT)	0.17 m/s (0.3)	0.60 m/s (0.5)	1.44	0.93	0.45	51 (26.8)	127 (66.8)	49 (25.8)	N/A	N/A
	0.00 m/s (0.3)	0.56 m/s (1.0)								
MAS 3; balanced sitting	3.0 (1.9)	4.4 (1.8)	0.72	0.71	0.50	58 (30.5)	19 (10.0)	4 (2.1)	22 (11.6)	65 (34.2)
	3.0 (4.0)	5.0 (2.0)								
MAS 4; sitting to standing	1.6 (1.7)	3.8 (2.4)	1.27	1.00	0.50	52 (27.4)	58 (30.5)	28 (14.7)	12 (6.8)	83 (43.7)
	1.0 (2.0)	5.0 (5.0)								
MAS 5; walking	0.96 (1.5)	3.1 (2.2)	1.43	1.10	0.50	61 (32.1)	115 (60.5)	48 (25.3)	3 (1.6)	35 (18.4)
	0.00 (2.0)	4.0 (5.0)								
MAS mobility: sum of items 3-5	5.6 (4.0)	11.3 (5.7)	1.42	1.16	0.92	32 (16.8)	11 (5.8)	3 (1.6)	0 (0)	19 (10)
	5.0 (6.0)	13.0 (9.0)								

*Abbreviations*: *SD* standard deviation, *IQR* interquartile range, *ES* Effect Size I, *SRM* Standardised Response Mean, *mES* median-based Effect Size, *Q3/7* 30th to 70th inter-percentile range, *10mWT* ten-metre walk test, *MAS* Motor Assessment Scale, *N/A* not applicable.

### Proportion of people for whom scores that did not change

A proportion of this group of stroke survivors did not change their scores on each of the measures. For example, 45.8% of participants did not change their Step Test score during the admission, 26.8% of participants did not change their 10mWT and 32.1% of participants did not change their MAS item 5 scores. The measure that detected change for the highest proportion of participants was the MAS mobility score (summed items 3–5), with only 16.8% of participants having no change in this score.

### Floor and ceiling effects

**Figure 1:**
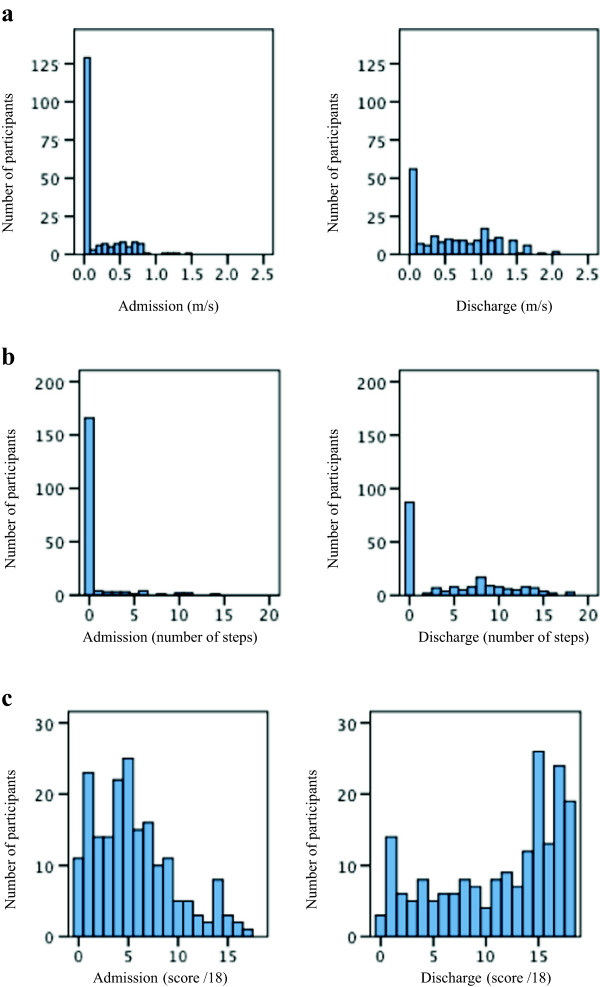
**Distribution of admission and discharge scores for the 10mWT, Step Test and MAS mobility scores. a**: Distribution of admission and discharge 10mWT scores, n = 190. **b**: Distribution of admission and discharge Step Test scores, n = 190. **c**: Distribution of admission and discharge MAS mobility scores, n = 190. Abbreviations: 10mWT = ten-metre walk test, m/s = metres/second, MAS = Motor Assessment Scale, MAS mobility = the summing of items 3, 4 and 5 of the Motor Assessment Scale.

The Step Test demonstrated a large floor effect on admission and discharge with 166 (88%) participants on admission and 87 (46%) participants scoring 0 (see Figure 1b). The 10mWT demonstrated a large floor effect on admission and discharge with 127 (67%) and 49 (26%) scoring 0 m/s respectively (see Figure 1a). These participants were unable to walk and consequently recorded a velocity of 0 m/s. The MAS item 3 (sitting) demonstrated a ceiling effect on discharge with 65 (34%) of participants scoring the maximum six points. The MAS item 4 (sit-to-stand) demonstrated a floor effect on admission with 58 (31%) of participants scoring 0 meaning they were unable to stand up with assistance. On discharge a ceiling effect was demonstrated with 83 (44%) participants who scored the maximum six points. MAS item 5 (walking) showed a large floor effect on admission with 115 (61%) participants scoring 0. The MAS mobility score (summed items 3 to 5) did not display floor or ceiling effects on admission or discharge measurement (see Figure 1c). Further details of the floor and ceiling effects for each item can be seen in Table 2.

## Discussion

This study investigated measures that are routinely used by physiotherapists on the study stroke unit, who then communicate the results to the clinical team in order to describe stroke survivors’ degree of improvement. The measures showed varied responsiveness to change in performance during inpatient care after stroke. Most measures displayed large responsiveness in at least one method of statistical analysis, apart from MAS item 3, which was consistently moderately responsive. As data were not normally distributed a median-based effect size was also calculated. This effect size showed consistently lower results than the other methods used (SRM 0.71-1.16 compared to mES 0.36-0.92). The MAS mobility was the most consistently responsive of the measurement tools investigated in this study with large responsiveness shown (mES 0.92).

There were a large proportion of stroke survivors whose scores on each of the measures did not change throughout the admission e.g. 46% of participants’ scores on the Step Test and 32% of scores on MAS item 5 did not change during the admission. The finding that a large proportion of stroke survivors’ scores did not change may be influenced by the study’s location and inclusion criteria. The study was located on a comprehensive stroke unit where acute and rehabilitation services are co-located. This means all stroke survivors access rehabilitation without selection of likely responders. Consequently all stroke survivors were included in the study regardless of initial post-stroke disability level or impairments. Thus, we have observed that even if stroke survivors have access to intensive rehabilitation a proportion will not improve their scores on these tests.

Furthermore, when designing this study we decided to use the lower of the Step Test scores obtained from stepping with each leg separately. This decision may have hampered the ability of the test to detect improvement and may also have contributed to the floor effect observed at both admission and discharge measurement. Similarly, with the 10mWT we assigned a value of zero to non-walkers. This may have contributed to the floor effect we observed at both admission and discharge. Clinically this test would not be used on those who cannot walk and would be responsive in those who can walk.

Both ceiling and floor effects were observed for most of the measures. These results are similar to those described in a stroke rehabilitation setting by Dean and Mackey [10] and English and colleagues [7]. The only measure to demonstrate no significant floor or ceiling effect at admission or discharge from the stroke unit was the MAS mobility score. This suggests that the method used (summing items 3–5) is a valuable way to detect change in performance in clinical settings similar to the study site. Generally, larger floor effects (more than 20% of participants at minimum score) were seen at admission and ceiling effects were only present for the discharge measures. For effective tracking of progress through rehabilitation, it could be argued that floor effects at admission are not a significant issue. There is room on the measure for improvement to occur and change in performance be demonstrated. However, floor effects at admission will limit the ability to distinguish between stroke survivors’ performance early in their stay e.g. for prediction of outcome.

This study found that the summed MAS displayed large responsiveness and of particular clinical significance, had no significant floor or ceiling effects. Furthermore, a summed measure such as this, has the ability to reflect stroke survivors’ mobility in general and not just their ability to step or walk in isolation. However, it has been argued that ordinal scales, such as the MAS items should not be summed, because they are not interval scales where all scores are of equal magnitude [20]. In other words, improving from a score of 1 to 2 on a MAS item may not be an equivalent improvement in performance to moving from a score of 3 to 4. We suggest that further investigation of the validity of the summing of lower limb MAS items is warranted to determine its robustness as a measurement tool. Previous research has investigated summing of the upper limb items of the MAS has demonstrated that the summation of these items is a reliable and valid measure of upper limb function after stroke [15].

It is important to note that this study occurred on a comprehensive stroke unit that provides intensive rehabilitation on a daily basis. As responsiveness is specific to the population being investigated [16], the results from this study may only be transferable to other comprehensive stroke units that provide similarly intense therapy levels.

The strength of this study was our recruitment method, relatively large sample size and high follow up rate for surviving participants. All consecutive stroke survivors admitted to the stroke unit were included regardless of disability or other factors. In addition, these consecutive stroke survivors were followed from the acute phase after their stroke to the end of inpatient rehabilitation. Consequently, we were able to observe a representative sample of stroke survivors throughout their hospital journey without risk of selection bias.

The measures investigated were not an exhaustive list of possible measures of aspects of motor performance. They were chosen for pragmatic reasons, as these were the measures that were regularly being used on the stroke unit. The decision to follow participants until discharge from hospital ensured an excellent follow up rate, however it did mean that participants’ discharge assessments were completed at different time points post stroke. Reassessment of participants at the same point in time e.g. at two months post stroke would have facilitated comparison with other studies e.g. the study by Veerbeek et al. investigating prognosis of gait after stroke [21]. In future studies, it would be on interest to investigate if certain measurement tools are responsive in particular types or subgroups of stroke survivors. For example, the 10mWT may be more responsive if only the ambulatory stroke survivors were considered in the analysis.

## Conclusion

In conclusion, this study demonstrated that the responsiveness for four of the five individual measures investigated was relatively large, but the ceiling and floor effects (at discharge, in particular) limit the utility of different measures in patients at the extreme ends of the functional spectrum. The only measurement tool to demonstrate large responsiveness as well as no marked floor or ceiling effects was the MAS mobility score (summed scores of items 3–5).

## Competing interests

The authors declare they have no completing interests.

## Authors’ contributions

CS initiated the study design. KScr collected and analysed data and drafted the manuscript. KSch participated in data collection and edited the manuscript. CS led the data analysis and edited the manuscript. All authors read and approved the final manuscript.

## Pre-publication history

The pre-publication history for this paper can be accessed here:

http://www.biomedcentral.com/1471-2377/14/129/prepub
